# Anthropogenic and Environmental Constraints on the Microbial Methane Cycle in Coastal Sediments

**DOI:** 10.3389/fmicb.2021.631621

**Published:** 2021-02-18

**Authors:** Anna J. Wallenius, Paula Dalcin Martins, Caroline P. Slomp, Mike S. M. Jetten

**Affiliations:** ^1^Department of Microbiology, Institute for Water and Wetland Research, Radboud University Nijmegen, Nijmegen, Netherlands; ^2^Department of Earth Sciences, Faculty of Geosciences, Utrecht University, Utrecht, Netherlands

**Keywords:** marine microbiology, methane oxidation, eutrophication, methanogenesis, sediment, climate change, greenhouse gases

## Abstract

Large amounts of methane, a potent greenhouse gas, are produced in anoxic sediments by methanogenic archaea. Nonetheless, over 90% of the produced methane is oxidized via sulfate-dependent anaerobic oxidation of methane (S-AOM) in the sulfate-methane transition zone (SMTZ) by consortia of anaerobic methane-oxidizing archaea (ANME) and sulfate-reducing bacteria (SRB). Coastal systems account for the majority of total marine methane emissions and typically have lower sulfate concentrations, hence S-AOM is less significant. However, alternative electron acceptors such as metal oxides or nitrate could be used for AOM instead of sulfate. The availability of electron acceptors is determined by the redox zonation in the sediment, which may vary due to changes in oxygen availability and the type and rate of organic matter inputs. Additionally, eutrophication and climate change can affect the microbiome, biogeochemical zonation, and methane cycling in coastal sediments. This review summarizes the current knowledge on the processes and microorganisms involved in methane cycling in coastal sediments and the factors influencing methane emissions from these systems. In eutrophic coastal areas, organic matter inputs are a key driver of bottom water hypoxia. Global warming can reduce the solubility of oxygen in surface waters, enhancing water column stratification, increasing primary production, and favoring methanogenesis. ANME are notoriously slow growers and may not be able to effectively oxidize methane upon rapid sedimentation and shoaling of the SMTZ. In such settings, ANME-2d (*Methanoperedenaceae*) and ANME-2a may couple iron- and/or manganese reduction to AOM, while ANME-2d and NC10 bacteria (*Methylomirabilota*) could couple AOM to nitrate or nitrite reduction. Ultimately, methane may be oxidized by aerobic methanotrophs in the upper millimeters of the sediment or in the water column. The role of these processes in mitigating methane emissions from eutrophic coastal sediments, including the exact pathways and microorganisms involved, are still underexplored, and factors controlling these processes are unclear. Further studies are needed in order to understand the factors driving methane-cycling pathways and to identify the responsible microorganisms. Integration of the knowledge on microbial pathways and geochemical processes is expected to lead to more accurate predictions of methane emissions from coastal zones in the future.

## Introduction

Methane is an important greenhouse gas, contributing to 16% of global warming (Ciais et al., 2013). Total methane emissions to the atmosphere are estimated at 500–600 Tg CH_4_ yr^–1^ with yearly fluctuations in sinks and sources. The recently measured atmospheric concentration (1,870 ppb in June 2020; Dlugokencky, 2020) is 2.5-fold higher than that for pre-industrial times (720 ppb). Anthropogenic activity is believed to be the main cause for this rapid increase of atmospheric methane (Ciais et al., 2013), as human activity is responsible for an estimated 50–75% of total methane emissions (Conrad, 2009). Oxidation by hydroxyl radicals is the main sink for methane in the atmosphere, accounting for 90% of all removal (Kirschke et al., 2013). Microbial methane production and oxidation, which occurs both aerobically and anaerobically by bacterial and archaeal species, regulates the amount of methane released into the atmosphere (Hanson and Hanson, 1996; Knittel and Boetius, 2009).

Methane is released from various sources including the Earth’s crust (thermogenic origin), incomplete combustion of fossil fuels and biomass (pyrogenic origin), and microbial metabolism (biogenic origin; Kirschke et al., 2013). Most biogenic methane is produced by microorganisms, mainly methanogenic archaea, as the last step in the breakdown of organic matter in anoxic environments, via the process of methanogenesis. Smaller amounts of methane are produced from methylphosphonates in oxic waters by cyanobacteria and other phytoplankton, and in the ocean by Thaumarchaea (Metcalf et al., 2012; Klintzsch et al., 2019; Bižiæ et al., 2020).

Methanogens are found both in natural and human-influenced systems, frequently where all electron acceptors other than CO_2_ are exhausted. Anthropogenic sources include rice fields, ruminant guts, landfills and sewage systems, which are ideal habitats for methanogens and emit significant amounts of methane into the atmosphere (Conrad, 2009). Natural methane sources include wetlands, other freshwater and marine systems, termites and thawing permafrost soils. Wetlands contribute almost to a third of overall global emissions, with estimates ranging from 80 Tg CH_4_ yr^–1^ to 280 Tg CH_4_ yr^–1^ (Bridgham et al., 2013). However, while oceans cover 70% of the Earth’s surface, they contribute only 1–2% of the global emissions (6–12 Tg CH_4_ yr^–1^; Weber et al., 2019). A significant portion of this, up to 75%, originates from coastal environments, which cover only a fraction of the total ocean area (Hamdan and Wickland, 2016). Degradation of organic matter in marine sediments produces large amounts of methane, but due to efficient removal by methane oxidation over 90% of this methane is microbially filtered and removed prior to emission (Knittel and Boetius, 2009).

In marine sediments, methanogens inhabit the deep anoxic layers where methanogenesis is the terminal step in organic matter degradation (Ferry, 1992). Archaeal methanogens use the enzyme complex methyl-coenzyme M reductase (MCR) to produce methane. Methanogens use a limited number of substrates, i.e., formate, acetate, hydrogen or methylated compounds (Reeburgh, 2007). The type of substrates used depends on the microorganisms present and the quality of the organic matter being degraded, which varies in different environments (e.g., LaRowe et al., 2020).

Above the methanogenic zone in marine sediments is a layer called the sulfate-methane transition zone (SMTZ) where upward diffusing methane and downward diffusing sulfate meet and are both removed (Barnes and Goldberg, 1976). At the start of the 21^*st*^ century, the pathways and responsible organisms for this removal were identified (Boetius et al., 2000; Orphan et al., 2001, 2002; Knittel and Boetius, 2009). In the SMTZ, methane is oxidized by anaerobic methane-oxidizing archaea (ANME) with sulfate-reducing bacteria (SRB) serving as the electron sink for the reaction (Knittel et al., 2018). ANME are phylogenetically related to methanogens and are divided into three clades (ANME-1, ANME-2 and ANME-3). Based on the Genome Taxonomy Database (GTDB), ANME-2 and ANME-3 belong to different *Methanosarcinales* clades, while ANME-1 is currently assigned to the Candidatus *Syntropharchaeia* class (Parks et al., 2020). All clades use a reverse methanogenesis pathway to oxidize methane, but some clades have been hypothesized to use the enzyme machinery for methane production as well (Lloyd et al., 2011). Members of the ANME-1 clade were recently identified as the main contributors to methane removal and potentially also methanogenesis in estuarine sediments, and the preferred metabolic process may be partly regulated by hydrogen concentrations (Kevorkian et al., 2020).

Sulfate-dependent anaerobic methane oxidation (S-AOM) by ANME consortia efficiently filters most of the upward diffusing methane in the SMTZ of deep marine sediments. The SRB can also couple dissimilatory sulfate reduction to organic matter degradation, and, given the sulfate concentration of 28 mM in seawater, they are the main microorganisms responsible for organic matter mineralization in these sediments (Jørgensen et al., 2019). As SRB have a high affinity for methanogenic substrates such as acetate and hydrogen, SRB can outcompete methanogens (Kristjansson et al., 1982; Schönheit et al., 1982). Therefore, methanogenesis usually does not occur above the zone of S-AOM but does dominate in deeper sediments where most, if not all sulfate, has been exhausted and still enough organic matter is present. However, cryptic methane cycling fueled by methylotrophic methanogenesis has been reported in sulfate-rich surface sediments of Aarhus Bay in Denmark (Xiao et al., 2018) and in the SMTZ at multiple sites in the Baltic Sea, where ANME-1 were hypothesized to mediate such activity (Beulig et al., 2019).

Although in most marine sediments with a well-defined SMTZ and steady ANME community methane removal is typically efficient (Knittel and Boetius, 2009), this is less so in many coastal sediments. One reason is that the SMTZ is generally located closer to the sediment-water interface, because of a lower salinity and associated lower sulfate concentrations. The smaller distance to the overlying water allows more methane to escape from the sediment through either diffusion or advection (Egger et al., 2018). Furthermore, the highly dynamic conditions in many coastal systems, i.e., linked to seasonal changes in temperature and primary production and/or variations in sedimentation rates, can impact the balance between methanogenesis and methane oxidation in the sediment. The key factors regulating methane cycling in coastal sediments are, however, not well understood (Hamdan and Wickland, 2016).

Besides S-AOM, various other pathways of methane oxidation may be quantitatively important in coastal sediments. In oxic surface sediment layers, aerobic methane-oxidizing bacteria (MOB) are expected to dominate (Rasigraf et al., 2017). MOB belonging to the phyla Proteobacteria and Verrucomicrobia use a particulate or soluble methane monooxygenase (pMMO; sMMO) to oxidize methane seeping through the SMTZ (Hanson and Hanson, 1996; Op den Camp et al., 2009). There is also accumulating evidence for anaerobic methane oxidation with nitrate, metal oxides and humic substances in freshwater and brackish coastal habitats (e.g., Raghoebarsing et al., 2006; Sivan et al., 2011; Haroon et al., 2013; Segarra et al., 2013; Egger et al., 2015b; Scheller et al., 2016; Martinez-Cruz et al., 2017; Valenzuela et al., 2017; Bai et al., 2019; van Grinsven et al., 2020). While bacteria of the NC10 candidate phylum named ‘*Candidatus* Methylomirabilis oxyfera’ can couple methane oxidation to nitrite reduction and nitric oxide dismutation (Ettwig et al., 2010), archaea belonging to the ANME-2 subclade ANME-2d (also known as family *Methanoperedenaceae*) can couple methane oxidation to reduction of nitrate (Haroon et al., 2013). Members of *Methanoperedenaceae* have also being identified as responsible organisms for methane oxidation coupled to iron and manganese reduction (Ettwig et al., 2016; Cai et al., 2018; Leu et al., 2020).

Coastal environments are vulnerable ecosystems that differ from open marine habitats by being more dynamic. Environmental conditions can change rapidly due to seasonal changes in temperature and salinity, in the composition of organic matter in terrestrial run-off, fluctuating rates of primary production and changes in hydrological conditions (Bauer et al., 2013). As the barrier between marine and terrestrial habitats, coastal sediments play an important role in the biogeochemical cycling of carbon, nitrogen, phosphorus, and metals (Sundby, 2006). Mineralization of organic compounds in coastal sediments is generally very rapid due to a high biodegradability of organic matter and a sufficient supply of electron acceptors, in addition to oxygen. Due to a complex network of metabolic processes, small changes in environmental factors may affect the redox zonation and biogeochemical functioning, including the methane cycle. Therefore, anthropogenic activity typically has a large impact on biogeochemical processes in coastal ecosystems. For example, eutrophication affects the redox state and microbial communities of coastal sediments (Doney, 2010).

Anthropogenically-induced nutrient inputs to coastal waters feed primary production in the form of intense algal spring blooms, which can result in bottom water oxygen depletion and high burial rates of organic matter (Diaz and Rosenberg, 2008). Additionally, climate change impacts hydrological conditions, which can lead to changes in bottom water salinity and/or water level. Global warming affects gas solubility, rates of chemical reactions and microbial activity (Doney, 2010). However, little is known about how these changes will influence element cycling in eutrophic coastal areas.

This review aims to summarize the current state of research on methane cycling in coastal sediments and the different factors driving methanogenic and methanotrophic processes. As anthropogenic eutrophication and global warming are increasing, coastal systems are severely impacted. Rapid burial of organic matter, changes in temperature and oxygen availability together with a high availability of alternative electron acceptors such as metal oxides and nitrate all alter the methane-filtering potential in coastal habitats. The impact of these changing factors is not well understood, and it is therefore difficult to estimate, let alone predict, future coastal methane emissions. However, we urgently need more accurate models to predict the role that different types of sediments have in increasing or decreasing methane emissions from coastal zones in the future.

## The Effect of Eutrophication and Climate Change on Sediment Biogeochemistry and Methane Fluxes

Eutrophication of coastal areas is of growing concern, as human activities cause major disturbances of coastal ecosystems. Land management practices such as agriculture, dam building, peatland draining and wetland removal alter the amount and content of organic carbon delivered to coastal areas (Bauer et al., 2013). Further, constant development of coastal areas may directly affect the methane cycling in estuaries, with sediments from estuaries subject to a higher intensity of human modification potentially emitting more methane into the water column (Wells et al., 2020). Anthropogenically-driven climate change results in higher average annual temperatures and induces hydrological and oceanographic variations, such as changes in water level and river flows, and might increase greenhouse gas emissions (IPCC, 2014). Flooding events in rivers have been shown to increase estuarine stratification and hypoxia (Kerimoglu et al., 2020) as well as methane emissions (Sieczko et al., 2016), with the composition of the terrestrial organic matter possibly determining which greenhouse gases are released (Schade et al., 2016). Increased hypoxia alters biogeochemical processes, with anaerobic pathways of organic matter degradation, including methanogenesis, gaining importance. Upon mild hypoxia, however, responses may be variable and difficult to predict (Foster and Fulweiler, 2019; LaRowe et al., 2020). Eutrophication can lead to an upward shift of the SMTZ (Crill and Martens, 1983), with past eutrophication potentially affecting rates of methanogenesis observed today (Myllykangas et al., 2020a).

### Influence of Sedimentation Rates on Coastal Methane Emissions

At the global scale, sedimentation rates, organic matter input, the depth of the SMTZ and the upward flux of methane to the sediment-water interface are related (Egger et al., 2018): the higher the sedimentation rate, the more organic carbon burial and the shallower the SMTZ because of increased methane production at depth. Hence, variations in sedimentation rate contribute to the difference in the average depth of the SMTZ in coastal and inner shelf sediments (≤2 m below the sea floor) when compared to sediments from more offshore areas (>4 m below the seafloor) (Egger et al., 2018). The shallower the SMTZ, the more chance of escape of methane from the sediment to the overlying water and, ultimately, to the atmosphere (Borges and Abril, 2012).

For example, elevated methane fluxes across the sediment-water interface due to high rates of sedimentation were reported for the Himmerfjärden estuary (Sweden) in the Baltic Sea (Thang et al., 2013). Porewater analysis at three sites in the estuary revealed a shallow SMTZ at 20 cm below the sediment surface, as defined by the depth of sulfate penetration (Thang et al., 2013). Overlapping sulfate and methane depth profiles indicate that the sediments at Himmerfjärden had a poor methane removal capacity in the SMTZ. Methane concentrations below the SMTZ were highest at the site closest to the shore, located directly downstream from a sewage treatment plant. At this site, the sedimentation rate and organic matter burial rates, were also highest (Table 1). This suggests that nutrients and organic matter leaking from the sewage treatment plant contributed to eutrophication in the nearby coastal areas. Organic carbon burial rates were, overall, relatively high at all stations. The high organic matter burial rates and shallow SMTZ allowed labile organic matter as well as reactive iron to be buried below the SMTZ. Active methanogenesis was observed in and below the SMTZ. Together, the weakened methane-filtering ability in the SMTZ, caused by the high sedimentation and organic matter burial rates, were suggested to increase methane emissions (Thang et al., 2013). A similar relationship between sedimentation rate and less efficient methane removal was described in a field study in Lake Grevelingen, a saline coastal reservoir suffering from eutrophication (Egger et al., 2016b). Porewater profiles combined with records of sedimentation and organic matter burial showed a link between eutrophication and rise in methane fluxes. The high rates of sedimentation and organic matter burial, when compared to many other coastal sediments (Table 1), are not unusual for coastal reservoirs and likely explain the shallow position of the SMTZ. The isotopic composition of upward diffusing methane in Lake Grevelingen sediment indicated that methanogenesis occurred within the SMTZ. Most of the methane bypassed the SMTZ and was likely either oxidized at the oxic surface of the sediment by aerobic methanotrophs or escaped to the overlying water (Egger et al., 2016b).

**TABLE 1:** Sedimentation rates and reported diffusive CH_4_ fluxes from sediments to the overlying water for selected coastal sediments.

**Location (water depth (m))**	**Sedimentation rate (cm yr^–1^)**	**Organic matter accumulation rate (mol C m^–2^yr^–1^)**	Diffusive CH_4_ efflux (mol m^–2^ yr^–1^)*	**References**
Lake Grevelingen (45)	13	91	0.2–0.8	Egger et al., 2016b
Himmerfjärden estuary station H5; downstream from sewage plant (25)	0.98	9.5	0.37	Thang et al., 2013
Himmerfjärden estuary station H3 (50)	0.82	9.3	0.25	Thang et al., 2013
Himmerfjärden estuary station H2 (30)	0.77	8.9	0.11	Thang et al., 2013
Average Inner shelf** (0–50)	0.56	7.8	0.21	Egger et al., 2018
Average Outer shelf** (50–200)	0.14	3.4	0.05	Egger et al., 2018

*Diffusive fluxes are calculated from porewater profiles and bottom water concentrations and do not include ebullition. **Based on literature review. For a recent overview of benthic release of CH_4_ in coastal systems, see Egger et al. (2016b).

Rapid sediment accumulation in coastal areas such as the Himmelfjärden estuary and Lake Grevelingen is associated with high organic matter input resulting from anthropogenic-driven eutrophication. The longer eutrophication persists, the more the redox zonation and biogeochemical functioning of the sediment may alter. Rapid sedimentation changes the depth of the SMTZ by bringing it closer to the sediment surface. This results in a shorter residence time of organic matter in the sulfate reduction zone, thereby leaving more substrate for methanogenesis below the SMTZ (Sundby, 2006; Dale et al., 2019).

The most common substrates for methanogens in coastal sediments are acetate (acetoclastic methanogenesis) and hydrogen (hydrogenotrophic methanogenesis; Crill and Martens, 1986). Little is known about the importance of methylotrophic methanogenesis in coastal ecosystems so far (Zhuang et al., 2017).

Methanogenesis is usually observed only in deeper sediments where all other electron acceptors have been exhausted, because SRB can outcompete methanogens on substrates such as acetate and hydrogen. Therefore, the SMTZ is usually a barrier for methane production. In many offshore marine sediments, sulfate-AOM in the SMTZ is so efficient that all methane is removed at the top of this zone (Reeburgh, 2007). Furthermore, methane could be produced in the SMTZ in addition to the layer below, due to a high abundance of methanogenic substrates. Combined with sulfate limitation, SRB can no longer outcompete methanogens for all the substrates in such a situation. In addition, methylated compounds such as dimethyl sulfide are non-competitive substrates and, therefore, methylotrophic methanogens may also be commonly present in the SMTZ (Mitterer, 2010).

In addition to the high abundance of substrates to support simultaneous sulfate reduction, AOM and methanogenesis, the SMTZ might also have a reduced ability to filter methane, regardless of the increased production. High sediment accumulation rates such as the one recorded at the Himmerfjärden estuary mean that the residence time of the microbial community in the SMTZ is only 20–30 years (Thang et al., 2013) and even shorter in Lake Grevelingen where the sedimentation rate is 10-fold higher (Egger et al., 2016b). The main organisms responsible for methane removal, ANME archaea, are known to be very slow growers (Knittel et al., 2018), and can possibly not establish enough biomass, as approximately 60 years are needed for ANME to create a steady-state AOM biomass (Dale et al., 2008a). Therefore, it is possible that the methane removal in these sediments is impaired, as the methane-oxidizing consortia cannot keep up with the rapid accumulation. The decreased ability to remove methane in the SMTZ, added to the increased methanogenic potential below this layer, strongly indicates that rapid sedimentation rates cause increased methane emissions from coastal sediments.

### Impact of Spreading Coastal Hypoxia on Sediment Processes

Hypoxia occurs in water bodies that are poorly ventilated and/or receive large inputs of organic matter (Middelburg and Levin, 2009). In these systems, hypoxia can be seasonal or more long lasting depending on the balance between the oxygen supply and demand. Human impact has extended the duration and spatial extent of hypoxia in many coastal systems. Following the widespread use of fertilizers since the 1950’s, many coastal areas have seen an introduction or expansion of hypoxia (≤2 mg O_2_ L^–1^; Breitburg et al., 2018). Examples of such areas are the Baltic Sea (Carstensen et al., 2014) and East China Sea (Wang et al., 2012). Hypoxia develops as eutrophication increases organic matter input, which promotes oxygen-consuming mineralization of organic matter. The degrading organic matter releases reduced compounds such as ammonium, and leads to release of Mn (II), Fe (II), and H_2_S, and their oxidation can deplete the remaining oxygen (Sundby, 2006). Higher average temperatures caused by global warming decrease the solubility of dissolved oxygen in coastal waters and may increase primary production and respiration. Consequently, oxygen is depleted faster in coastal waters (Diaz and Rosenberg, 2008). The longer the hypoxia remains, the larger the consequences are for the microbial and benthic community as well as the biogeochemical cycling of elements in the sediment (Middelburg and Levin, 2009).

**FIGURE 1:**
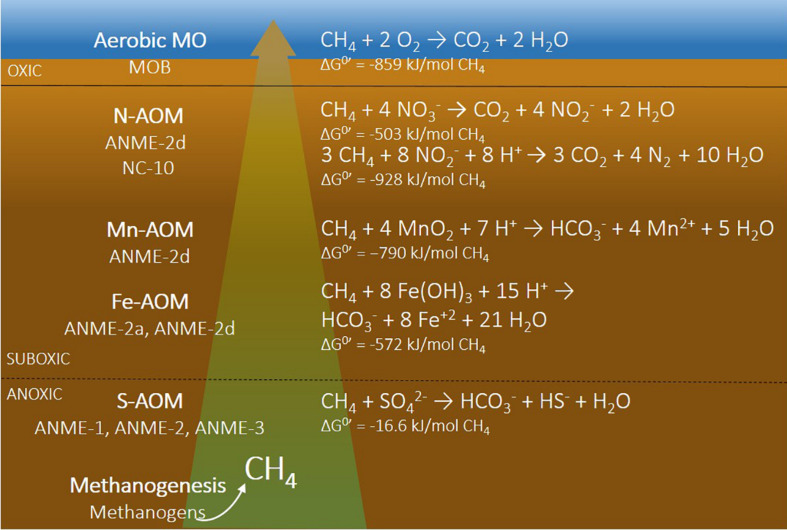
Schematic presentation of microbial processes and microorganisms **(left)** and reaction equations with different electron acceptors **(right)** involved in methane cycling. N-AOM can couple either nitrate (ANME-2d) or nitrite (NC-10) to methane oxidation. MO, methane oxidation; N-AOM, nitrate/nitrite-dependent anaerobic oxidation of methane; Mn-AOM, manganese-dependent anaerobic oxidation of methane; Fe-AOM, iron-dependent anaerobic oxidation of methane; S-AOM, sulfate-dependent anaerobic oxidation of methane. References for the Gibbs free energies are the following: aerobic MO (Regnier et al., 2011), N-AOM (In’ t Zandt et al., 2018), Mn-AOM and Fe-AOM (Sturm et al., 2019), and S-AOM (Meulepas et al., 2010).

A decrease in bottom water oxygen alters the pathways of organic matter mineralization and methane cycling in sediments. In sediments covered by oxic bottom waters, various electron acceptors are typically available which can be used for organic matter mineralization in the following sequence (based on the Gibbs free energy yield): oxygen, nitrate (NO_3_^–^), manganese [Mn(IV)], iron [Fe(III)], sulfate (SO_4_^2–^), and finally CO_2_, for methanogenesis (Figure 1; Froelich et al., 1979; LaRowe et al., 2020). Many of these electron acceptors are also involved in additional redox reactions, including methane oxidation (Figure 1) and cryptic cycling of Fe and S. In sediments overlain by hypoxic and anoxic bottom waters, however, the redox zonation will become more compressed and, especially upon persistent anoxia, may include only the zones of sulfate reduction and methanogenesis. Upon prolonged periods of bottom water anoxia, the mineralization of organic matter in the sediment may not be as effective (Hartnett et al., 1998; Middelburg and Levin, 2009). Seasonal bottom water hypoxia, however, could accelerate organic matter conversion, as studies have shown that alternating oxic-anoxic cycles were most efficient for mineralization (Sundby, 2006).

Bioturbation may also alter the sediment redox zonation. Macrofauna can transfer fresh organic matter and electron acceptors from surface sediments and the overlying seawater to deeper sediment layers, thereby changing the microbial community structure and composition and potentially accounting for the dominance of bacteria over archaea in marine sediments (Chen et al., 2017; Deng et al., 2020). In the Baltic Sea, macrofauna may increase methane release (up to eightfold) from sediments in relation to macrofauna-devoid sediments, potentially accounting for an estimated 9.5% of total emissions from this marine ecosystem (Bonaglia et al., 2017). The hypothesized mechanism for macrofauna-induced benthic methane efflux was a combination of a flush-out effect and methanogen symbionts of macrofauna. However, this does not exclude the hypothesis that macrofaunal activity could also enhance methane oxidation due to the introduction of electron acceptors into deeper sediment layers (Kogure and Wada, 2005). On the other hand, hypoxia and anoxia often disperse or completely eliminate macrofauna from coastal marine sediments, promoting the further accumulation of reduced compounds, including sulfide (Middelburg and Levin, 2009). Sulfide might not affect methanogenic activity, but could be toxic to ANME archaea (Timmers et al., 2015), potentially decreasing anaerobic oxidation of methane. The complex mechanisms and impacts of the presence or absence of macrofaunal activity remain to be fully elucidated.

A direct link between seasonal hypoxia and higher methane fluxes was recently observed in the eutrophic Chesapeake Bay, United States (Gelesh et al., 2016). Upon oxygen depletion in the bottom water following the algal spring bloom, benthic methane release increased. By the end of summer, methane was detected across the stratified water column up to the surface waters and was thus likely released to the atmosphere. However, once oxygen was reintroduced in the bottom waters, methane concentrations decreased significantly, and disappeared to background levels during winter. One possibility for the observed methane trend is that methanogenesis rates increased in the anoxic sediments, and the anaerobic oxidation was too slow to respond. Another explanation for the sudden decrease in methane levels during summer is the transport of dissolved methane from elsewhere, due to seasonal storms or algal blooms, to the measurement site (Gelesh et al., 2016). However, as biogeochemical processes regulating the sediment methane cycle are very complex and affected by changes in many factors such as sedimentation rate, temperature and availability of electron acceptors in addition to spatial and temporal depletion of oxygen, more field studies are needed to establish a connection between hypoxia and methane emissions.

### Climate Change-Induced Rising Temperatures and Sediment Biogeochemistry

Human-driven climate change is increasing average seasonal temperatures globally. Since pre-industrial times, global temperature has risen 1°C, with much greater increases locally, especially in polar and sub-tropical coastal zones (IPCC, 2014). In coastal areas, increasing temperatures have been linked to increases in greenhouse gas emissions, such as elevated sediment-water fluxes of methane in coastal waters of Belgium (Borges et al., 2019) and Baltic Sea (Humborg et al., 2019) during a heatwave in 2018.

Local methane emissions may also increase due to long-term warming, as was demonstrated in artificial freshwater ponds with 4°C warming over a decade (Zhu et al., 2020). The over twofold increase in emissions was linked to disproportionate increase in methanogenesis activity, in comparison to other processes. Methanogenesis is known to strongly depend on temperature (Westermann et al., 1989; Schulz et al., 2006) and many methanogens may have a significantly higher optimum temperatures than their *in situ* habitat (Blake et al., 2020). Thus, a warming climate may favor methanogens and increase their activity, but the potential increase of methane production is difficult to link directly to higher emissions. Notably, Yvon-Durocher et al. (2014) used meta-analyses to show that the temperature dependence of methane production in natural ecosystems is higher than that of other carbon-cycling processes with a 4:1 ratio for CH_4_:CO_2_, resulting in potentially relatively higher global methane emissions in a warmer climate.

Methanogenesis using different substrates may also respond differently to rising temperature. Long-term warming shifted a freshwater community toward hydrogenotrophic methanogenesis, and hydrogen was favored over acetate also in incubation studies (Zhu et al., 2020). Similarly, laboratory incubations of coastal sediments from Gulf of Mexico showed the strongest effect of temperature on hydrogenotrophic methanogenesis, and methylotrophic methanogenesis were much less affected (Zhuang et al., 2018). These studies suggest that the different methanogenic clades inhabiting the sediments might determine the level of impact that temperature plays on increasing methane production rates.

Temperature dependence of methane oxidation is less understood. ANME archaea have mainly been characterized in cold, high-pressure environments such as deep sea vents (Boetius et al., 2000) where methane dissolves easier. Studies show that the optimal AOM rates vary between ANME clades, but are within a narrow range of 5–25°C (Bhattarai et al., 2018; Nauhaus et al., 2005), therefore suggesting that their activity would not increase significantly in warmer temperatures. However, more studies on the effect of temperature on AOM are needed to know whether anaerobic oxidation of methane is equally affected by global warming as methanogenesis, since this will determine the strength of the positive feedback loop of climate warming and increases in atmospheric methane concentrations.

## Eutrophication Alters the Redox Zonation in Coastal Sediments and Could Support Alternative Electron Acceptors for AOM

In freshwater environments with non-detectable amounts of sulfate, AOM has been linked to metal oxide, nitrate and nitrite reduction (Raghoebarsing et al., 2006; Segarra et al., 2013; He et al., 2016). In coastal areas with rapid rates of sedimentation and a decreased salinity, sulfate only penetrates the surface layers a few centimeters, and a large amount of reactive organic matter and metal oxides can be buried below the SMTZ (Egger et al., 2015a, 2016b; Rooze et al., 2016; Rasigraf et al., 2020). Run-off of nitrogen products from agricultural fertilizers also deliver more reactive nitrogen products to the coastal waters (Jickells, 1998). Therefore, anthropogenically-induced eutrophication might create conditions to supply AOM with alternative electron acceptors and these processes might become more important to understand the future in coastal areas affected by human activity. As the methane-removing efficiency is often compromised in coastal areas due to rapid sedimentation rates and high organic matter load (Thang et al., 2013; Egger et al., 2015b), it is important to understand how these alternative AOM processes contribute to methane removal.

### Metal Oxides Buried Below the SMTZ Could Serve as Electron Acceptors for AOM

In marine sediments, ANME-1, ANME-2 and ANME-3 have been linked to S-AOM in the SMTZ and are considered key microorganisms involved in methane removal (Knittel et al., 2018). In freshwater sediments where sulfate concentrations are low to non-existent, AOM may be coupled to more energetically favorable electron acceptors such as oxides of iron (Fe-AOM) and manganese (Mn-AOM; Cai et al., 2018). Alternatively, in such environments, manganese and iron oxides could lead to the oxidation of reduced sulfur species to sulfate, sustaining S-AOM by a syntrophic consortium of *Methanoperedenaceae* (ANME-2d) and sulfate-reducing *Desulfobulbaceae* (Su et al., 2020). Due to a high salinity and, hence, high availability of sulfate in marine sediments, iron oxides are usually not available as electron acceptors in the SMTZ, as they react with dissolved sulfide formed from sulfate reduction (Reeburgh, 2007). However, the possibility for Fe-AOM and Mn-AOM in marine settings was shown already a decade ago (Beal et al., 2009), but direct evidence for this process and details on the responsible microorganisms are still scarce.

Genomic and transcriptomic analysis of a freshwater sediment enrichment culture identified ANME-2d clade members as being responsible for coupling methane oxidation to the reduction of iron and manganese oxides (Cai et al., 2018; Leu et al., 2020), but these organisms are yet to be connected to potential Fe-AOM and Mn-AOM in marine sediments. Interestingly, also aerobic Proteobacterial methanotrophs were recently reported to be capable of coupling methane oxidation to metal reduction under hypoxia (Zheng et al., 2020). Furthermore, contradictory to previous knowledge, methanogens may benefit from iron oxides (Kato and Igarashi, 2019) and have been suggested to play a role in Fe-AOM (Elul et al., 2020) and be able to switch between methanogenesis and iron reduction in varying conditions (Sivan et al., 2016). These findings reveal the complexity of iron and methane cycling in sediments, highlighting the importance of unraveling the responsible microorganisms and biogeochemical pathways used.

Egger et al. (2015b) provided geochemical evidence for Fe-AOM in brackish coastal sediments of the Bothnian Sea from porewater profiles and laboratory incubations. Sediments in this deep part of the Bothnian Sea (214 m) are characterized by a shallow SMTZ, which is attributed to high inputs of organic matter and rates of sedimentation (Table 1). Below the SMTZ, high concentrations of iron oxides and dissolved Fe^2+^ were observed, as well as indications for active methanogenesis, based on isotopically depleted CH_4_. Slurry incubations of methanogenic layers with ^13^C-CH_4_ and iron oxides in the absence of sulfate showed increased ^13^CO_2_ production compared to those supplied only with methane, suggesting sulfate-independent AOM. This suggests Fe-AOM can occur in sediments where iron oxides and reactive organic matter are buried below the SMTZ. The potential controls on Fe-AOM at this site were explored further by Rooze et al. (2016) using a transient reactive transport model. The results confirm that increased organic matter input over several decades can lead to increased rates of methanogenesis and iron reduction, and a shoaling of the SMTZ. Inputs of Fe oxide and bottom water sulfate concentrations are also major controls on Fe-AOM rates, with sulfate inhibiting both methanogenesis and Fe-AOM.

Even though the ANME-2d clade has been linked to Fe-AOM and Mn-AOM in enrichment cultures isolated from freshwater sediments (Ettwig et al., 2016; Cai et al., 2018; Leu et al., 2020), very little is known about the microorganisms involved and their abundance and significance in other environments. Aromokeye et al. (2020) enriched a different subclade of ANME-2; ANME-2a, from methanic marine sediments of the Helgoland Mud area in the North Sea in incubations with iron oxides, whereas ANME-2d reads were not detected in the sediments. The incubations showed active Fe-AOM in the absence of sulfate, albeit at a rate of only 2% of the S-AOM rate recorded in the SMTZ (Table 2). In addition, 16S rRNA gene reads of dissimilatory iron-reducing bacteria increased in relative abundance in the same incubations. While active manganese reduction was seen in the porewater profiles, incubations amended with manganese oxides showed no active methane oxidation (Aromokeye et al., 2020).

**TABLE 2:** Comparison of estimated Fe-AOM and S-AOM rates from selected coastal sediments.

**Location**	**Fe-AOM rate (μmol CH_4_ cm^–3^ yr^–1^)**	**S-AOM rate in SMTZ (μmol CH_4_ cm**^–^**^3^ yr**^–^**^1^)**	**Method**	**References**
North Sea	0.035	2.0	Laboratory incubations	Aromokeye et al., 2020
Bothnian Sea (US5B)	1.3	–	Laboratory incubations	Egger et al., 2015a
Bothnian Sea (US5B)	0.08	0.79	Reactive transport model	Rooze et al., 2016
Bothnian Sea Öre estuary (NB8)	0.15	9.5	Reactive transport model	Lenstra et al., 2018; Rasigraf et al., 2020
Dover Bluff salt marsh	1.4	2.4	Laboratory incubations	Segarra et al., 2013
Black Sea	1.46*10^–5^	0.073	Reactive transport model	Egger et al., 2016a
Baltic Sea, Bornholm Basin	9.1*10^–4^	0.029	Reactive transport model	Dijkstra et al., 2018; Ash et al., 2019
Baltic Sea Landsort Deep	1.1*10^–3^	0.27	Reactive transport model	Egger et al., 2017

In another study, the microbial community composition in the Bothnian Sea, ANME-2a abundance was also linked to the presence of methane and iron oxides (Rasigraf et al., 2020). However, regardless of the parallel enrichment of iron-reducing bacterial taxa with ANME-2a (Aromokeye et al., 2020), it is not clear whether ANME-2a can oxidize methane independently, possibly via an extracellular electron acceptor, or whether they need a bacterial partner similar to the SRB in S-AOM. Incubation or enrichments fed with artificial electron acceptors, or transcriptomic profile of both bacterial and archaeal species could help to gain more insight into the physiology of Fe-AOM.

The evidence for Mn-AOM in coastal sediments is less clear. Although no indication of Mn-AOM was observed in Helgoland Mud sediment incubations (Aromokeye et al., 2020), Mn-AOM was recorded in incubations of sediments from deep methane seeps (Beal et al., 2009), coastal freshwaters, and brackish wetlands (Segarra et al., 2013). These differences could be due to disparities in the microbial community composition. Sulfate reduction was deliberately inhibited only in the Helgoland Mud incubations, indicating that observed Mn-AOM rates in other studies could have resulted from S-AOM if sulfate was available. Recently, Leu et al. (2020) showed active Mn reduction coupled to methane oxidation in a bioreactor enriched with members of the ANME-2d clade. Hence, further insight is needed in the potential role of manganese oxides as electron acceptors in AOM, the ANME clades capable of Mn-AOM, and the environmental factors favoring Fe-AOM and Mn-AOM in coastal and freshwater sediments.

**FIGURE 2:**
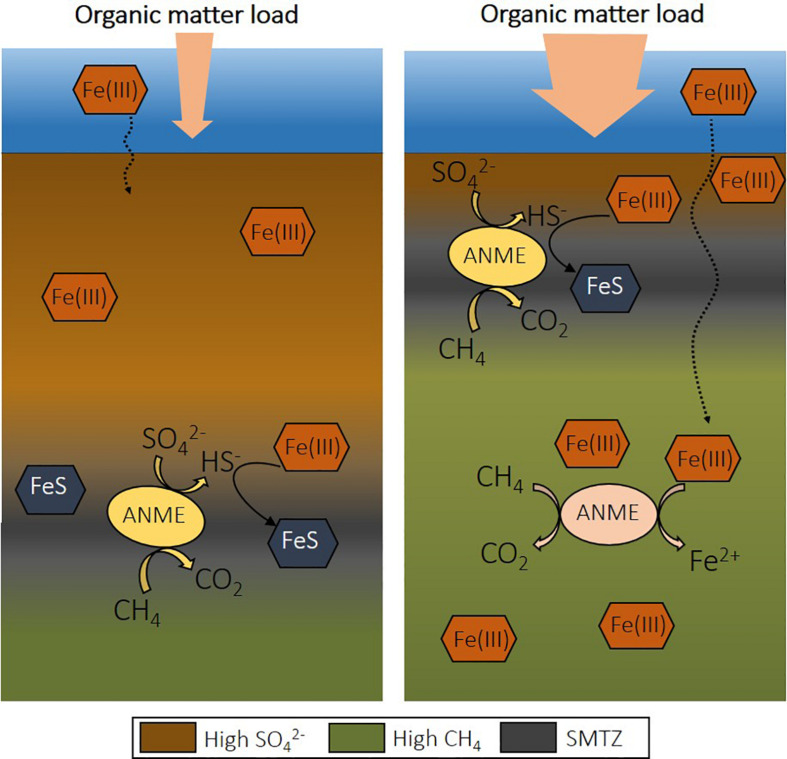
The effect of eutrophication on the depth of the sulfate-methane transition zone (SMTZ) in coastal sediment and the potential for Fe-AOM. Upon increase of organic matter load, the depth of sulfate penetration decreases due to organic matter degradation coupled to sulfate reduction. This shifts the SMTZ upward and allows the presence of iron oxides at deeper depths, creating a niche for Fe-AOM. Fe-AOM, iron-dependent anaerobic oxidation of methane; S-AOM, sulfate-dependent anaerobic oxidation of methane.

The rates recorded for Fe-AOM are significantly lower than S-AOM, accounting for less than 5% of methane oxidation in coastal marine sediments (Table 2). However, eutrophication causes several changes that might favor Fe-AOM in deep methanic sediments. If the SMTZ is moved upward and the organic matter load is high, iron oxides spend less time in this layer and can be buried below the SMTZ before reacting with dissolved sulfide (Figure 2; Rooze et al., 2016). Over time, iron oxides will become abundant and may serve as electron acceptors for AOM (Egger et al., 2015b). Iron oxides may also be more available for Fe-AOM in sediments of shallower coastal waters, as is suggested with a minimum 10-fold difference in iron reduction rates determined via reactive transport models from offshore sites (Table 2). Potentially, in deeper sediments the more reactive iron oxides are already exhausted, or estimated Fe-AOM rates correspond instead to dissimilatory iron reduction.

An increased Fe input upon the expansion of eutrophication-driven hypoxia is, for example, recorded in sediments of the open Baltic Sea (Reed et al., 2016). Changes in land-use and/or climate may also lead to episodic increased terrestrial inputs of organic matter and Fe oxides to coastal sediments (Lenstra et al., 2018). As a consequence, iron oxides could be available over a wide depth interval. As the SMTZ in coastal areas is usually located at a shallow depth, a relatively wide Fe-AOM zone could increase the role of this alternative methane oxidation pathway.

### Nitrate and Nitrite as Electron Acceptors for AOM

Methane oxidation coupled to nitrate/nitrite reduction (N-AOM) by both bacterial and archaeal species has been observed in freshwater sediments (Raghoebarsing et al., 2006; Luesken et al., 2011; Shen et al., 2015), and could also play a role in removing methane in coastal marine sediments (Shen et al., 2016; Wang J. et al., 2019) and marine oxygen minimum zones (Padilla et al., 2016; Thamdrup et al., 2019). The N-AOM *Candidatus* Methylomirabilis oxyfera bacteria can couple methane oxidation to nitrite reduction and nitric oxide dismutation (nitrite-AOM) using intracellularly produced oxygen and the aerobic methane oxidation pathway (Ettwig et al., 2010). The archaeal species belonging to the ANME-2 subclade ANME-2d (also known as family *Methanoperedenaceae*) can couple methane oxidation to reduction of nitrate (Haroon et al., 2013). In contrast to S-AOM, nitrate-dependent AOM (nitrate-AOM) does not require syntrophy. However, *Candidatus* Methanoperedens nitroreducens is often found in a community together with nitrite scavengers such as anammox and N-AOM bacteria (Haroon et al., 2013; Vaksmaa et al., 2017; Gambelli et al., 2018) to prevent inhibition by nitrite. Moreover, dissimilatory nitrate reduction to ammonium by *Methanoperedenaceae* has been recently reported to suffice as ammonium supply for anammox activity in a bioreactor enrichment culture (Nie et al., 2020). Several genomes of Methylomirabilis bacteria and ANME-2d archaea have been sequenced (Ettwig et al., 2010; Haroon et al., 2013; Arshad et al., 2015; Berger et al., 2017; Vaksmaa et al., 2017; Versantvoort et al., 2018) and recently the major protein complexes involved in the central metabolism of both microorganisms have been elucidated (Versantvoort et al., 2019; Berger et al., 2021).

Despite rapidly emerging new studies on N-AOM, the factors affecting the rates of nitrate- and nitrate-AOM and abundance of the responsible organisms are not well understood (Welte et al., 2016), and even less is known about the actual contribution of N-AOM to methane removal in coastal sediments. As anthropogenic eutrophication brings more organic matter to coastal sediments and increases nutrient concentrations in bottom waters, substantial amounts of nitrate and nitrite may become available as electron acceptors (Kraft et al., 2014). Increases in global eutrophication might therefore make these processes more important in methane removal, especially in sediments where sulfate concentrations are generally low and S-AOM is further compromised by shoaling of the SMTZ.

Significant rates of N-AOM were observed in coastal wetland and estuary sediments in China, with high concentrations of nitrate and nitrite in the sub-surface sediment (He et al., 2019; Wang J. et al., 2019). Molecular analysis revealed an abundant NC10 bacterial community in the layers below the oxygen penetration zone and the recorded rate for nitrite-AOM was faster than the S-AOM rate in deeper layers. Most of the methane was removed at the same depth where NC10 sequences were present, indicating their important role as a methane sink in these sediments. However, ANME-2d were also abundant and present in all samples showing N-AOM activity by isotopic tracing of ^13^CO_2_, indicating a potential niche separation of microorganisms performing nitrite- and nitrate-AOM.

The depth distribution of *Ca.* M. oxyfera and ANME was hypothesized to result from better oxygen tolerance of *Ca.* M. oxyfera bacteria. However, oxygen exposure studies showed that oxygen as low as 2% (vol/vol) for *Ca.* M. oxyfera (Luesken et al., 2012) and 5% for archaea *Ca.* Methanoperedens nitroreducens (Guerrero-Cruz et al., 2018) can cause oxidative stress and a rapid decrease in methane oxidation potential. Nevertheless, genomes of both organisms indicate the potential to counteract exposure to oxygen. Therefore, other biogeochemical factors probably play a role in the observed depth profile of nitrate- and nitrite-AOM, but further studies are needed to understand the spatial and temporal dynamics of N-AOM in coastal sediments with fluctuating oxygen concentration.

In general, N-AOM has been shown to be downregulated by the presence of sulfate (Wang J. et al., 2019) and salinity (Shen et al., 2016). In contrast, temperature, ammonia and nitrate were positively correlated to N-AOM. This could imply that regardless of the inhibiting effect of sulfate, N-AOM activity will increase in coastal areas upon an increase in nutrient load as well as higher temperatures. Curiously, S-AOM has been shown to be inhibited by exogenous nitrogen addition (Zhang et al., 2020), emphasizing the potential role of N-AOM in eutrophic sediments. Furthermore, N-AOM is energetically more favorable than S-AOM, and therefore might have a higher methane removal potential if the organisms are able to grow faster than S-AOM ANME archaea (Wang J. et al., 2019). However, more information on the community structure and methane oxidation rates under different conditions is necessary to assess how eutrophication affects N-AOM in coastal sediments and its contribution to methane removal.

## Recent Advances and Challenges in the Identification and Characterization of Methane-Cycling Microorganisms in Coastal Marine Sediments

The discovery of anaerobic methanotrophs capable of converting methane with electron acceptors other than sulfate has brought us a step closer to understanding and predicting methane cycling in different coastal environments. However, the methane cycle is only a small though important fraction of an entire network of microbial metabolism involved in the decomposition of organic matter, which drives element cycling in sediments (Jørgensen et al., 2019; LaRowe et al., 2020). Geochemical porewater profiles do not reveal the complex active microbial network in sediments, as locations with a similar redox zonation might harbor an entirely different microbiome and active metabolic pathways (Grossart et al., 2020). Therefore, understanding the microbial community composition of sediments, including their metabolic potential and interactions with the abiotic environment is a major challenge.

The advances in high throughput gene sequencing and metagenomic analysis in the last decade have enabled numerous studies on the microbial community composition of diverse aquatic environments using pyro-, 16S rRNA gene, and metagenome sequencing (reviewed in Grossart et al., 2020). Yet, linking specific activity to the genes and microorganisms present is difficult. Fortunately, the database of metagenomic sequences extracted from different sediments is growing, and linking this genome information to geochemical data is increasing our understanding of factors influencing the sediment microbiome involved in biogeochemical processes such as nitrogen cycling (Rasigraf et al., 2017; Reyes et al., 2017) and iron reduction (Reyes et al., 2016; Vuillemin et al., 2018).

### Anaerobic Methanotrophs

Metagenomic approaches have revealed the diversity and distribution of methane-cycling microbes such as the ANME archaea (Table 3). The ANME clades identified so far appear to prefer different conditions and substrates and are often linked to specific environments. For example, ANME-2d, which are capable of Fe-AOM/Mn-AOM, are usually found in freshwater sediments (Cai et al., 2018; Leu et al., 2020), whereas ANME-2a are observed in marine environments (Aromokeye et al., 2020). ANME-1 are traditionally classified as a marine clade performing S-AOM together with SRB (Knittel et al., 2018), but related sequences have been found in ferruginous freshwater sediments poor in sulfate, hinting at potential for Fe-AOM (Vuillemin et al., 2018). Another explanation could be that the detected ANME-1 cells are involved in methanogenesis as suggested in other studies (Lee et al., 2016; Kevorkian et al., 2020). Targeting iron-reducing microorganisms and/or genes, has increased the understanding of potential habitats for Fe-AOM activity. Furthermore, new bioinformatic tools are arising for screening of ubiquitous genes such as those involved in iron cycling (FeGenie; Garber et al., 2020), possibly enabling identification of novel taxa involved in iron reduction in coastal sediments. Similarly, genomic approaches have identified separate niches for N-AOM organisms showing that nitrate-reducing ANME archaea often prefer deeper sediments, whereas nitrite-reducing *Ca.* M. oxyfera-like bacteria are found closer to the oxic zone, regardless of the distribution of their substrate (He et al., 2019; Xie et al., 2020).

**TABLE 3:** Distribution of ANME archaea clades and their suggested metabolism in marine and freshwater sediments as found in metagenomic studies.

**Location (mbsf)**	**Sediment characteristics**	**Method**	**Detected ANME clades**	**Suggested AOM-pathway**	**References**
Gulf of California Guaymas basin hydrothermal sediment (2011)	Hydrothermal, hydrocarbon rich sediment; SMTZ/SR; 4–6, 8–11 cm deep	Metagenomics and metatranscriptomics	ANME-1, ANME-2c	S-AOM; multi-carbon alkane oxidizing archaea	Dombrowski et al., 2017; Wang Y. et al., 2019
Gulf of Mexico, cold seep (>1,000)	Biogenic methane seep; Thermogenic methane seep, top layer (0–15 cm)	Metagenomics, metatranscriptomics, 16S rRNA gene sequencing	ANME-1(b) ANME-2c	SRB-independent S-AOM S-AOM in syntrophy with SRB	Vigneron et al., 2017, 2019 Vigneron et al., 2019
South China Sea, Dongsha area (1,024)	High methane flux (methane clathrates below); above SMTZ; 10–330 cm deep	Metagenomics, 16S rRNA gene sequencing	ANME-1b	S-AOM and Fe-AOM based on presence of Fe(III)/Fe(II) and Fe-reduction genes (supported by geochemical data)	Li et al., 2019
East Sea of Korea, Ulleung Basin (>1,000)	SMTZ, methane hydrate-bearing sediment	Metagenomics	ANME-1b	S-AOM	Lee et al., 2016
Nyegga cold seep, Norwegian Sea (746)	Methane seep; top 15 cm	Metagenomics and metaproteomics	ANME-1	SRB-independent S-AOM	Stokke et al., 2012
Bothnian Sea (Öre estuary 21–33; offshore 214)	Methanic; rich in Fe-oxides; below SMTZ (16S first 50 cm)	Metagenomics (Öre estuary) 16S rRNA gene sequencing (all)	ANME-2a	Fe-AOM; ANME-2a correlates to methane and Fe-oxides below SMTZ	Rasigraf et al., 2020
Baltic Sea, Aarhus Bay (27)	SMTZ	Metagenomics	ANME-1	S-AOM and methanogenesis (cryptic CH_4_ cycling)	Beulig et al., 2019
Marine Lake Grevelingen, the Netherlands (45)	SMTZ	16S rRNA gene sequencing, batch incubations	ANME-3	SRB-independent S-AOM	Bhattarai et al., 2017
Pojo Bay estuary, Finland (7–34)	SMTZ and below	Metagenomics, 16S rRNA gene sequencing, batch incubations	ANME-2a/b	Fe-AOM	Myllykangas et al., 2020b
Lake Towuti, Indonesia, ferruginous lake (60; 200)	Top 50 cm	Metagenomics, 16S rRNA gene sequencing	ANME-1	Unclear – Fe-AOM?	Vuillemin et al., 2018
Gold Creek Reservoir, Australia (10)	Enrichment culture of top sediment layer	Metagenomics, metatranscriptomics, 16S rRNA gene sequencing (enrichment culture)	ANME-2d	Fe-AOM	Cai et al., 2018
Ferruginous terrestrial mud volcano, Taiwan (0)	0–160 cm depth profile	Metagenomics, 16S rRNA gene sequencing	ANME-2a	Fe-AOM (possibly with a synthrophic bacterial partner)	Tu et al., 2017

Metagenomic approaches can be used to gain knowledge of the metabolic potential of unclassified or poorly known microbes. Rasigraf et al. (2020) combined porewater profiles, 16S rRNA amplicon sequencing and functional gene analysis of metagenome-assembled genomes (MAGs) to study the metabolic potential and microbial community composition linked to the geochemical characteristics of Bothnian Sea sediments. The dominant bacterial and archaeal taxa followed the redox zones at the sampled sites, and ANME-2a was found to directly correlate with the presence of methane and iron oxides at two sites. However, despite this similarity in redox zonation, the microbial community composition at the sites differed noticeably. For example, an abundant community of aerobic methanotrophs was observed in the surface at the most offshore site. Dominant taxa involved in organic matter mineralization at each site also indicated that the active biogeochemical processes were different. To analyze the metabolic potential, MAG construction and functional gene analysis targeting main metabolic pathways for S, N, and C cycling was performed on one methanogenic sediment layer. The characterization of the most dominant microbes complemented the geochemical and phylogenetic data and revealed a vast metabolic potential adapted to low availability of substrates and anoxia. Main pathways for all element cycling could be identified, such as denitrification for N-reduction and three different pathways for methanogenesis (Rasigraf et al., 2020). However, a metagenomic profile of the microbiome is only a snapshot in time and fails to reveal the active metabolisms. To link a metabolic process with a gene or a group of microbes, metatranscriptomic and -proteomic methods preferably at the single cell level would give a more accurate indication. Metatranscriptomic methods reveal the transcribed genes in the community and help identifying microorganisms involved in methane cycling, as was done for ANME-2a capable of Mn-AOM and Fe-AOM (Cai et al., 2018; Leu et al., 2020), and N-AOM activity in ANME-2d (Haroon et al., 2013). They are also critical when studying the factors affecting gene expression and methane-oxidizing activity in different environments (e.g., Narrowe et al., 2019; Vigneron et al., 2019). Finally, metaproteomics, the analysis of all expressed proteins in a community, is rising as an important method to unravel the physiology of microbes involved in biogeochemical cycling in coastal sediments and the influence of environmental factors on these processes (Saito et al., 2019; Grossart et al., 2020).

### Methanogens

Methanogenic archaea are better characterized than ANME, as there are several isolated representatives of different taxonomic groups. Metagenomic studies are rapidly adding new groups to the canonical methanogenic orders (i.e., *Methanococcales*, *Methanopyrales*, *Methanobacteriales*, *Methanosarcinales*, *Methanomicrobiales*, and *Methanocellales*), such as *Methanomassiliicoccales*, *Methanofastidiosa*, and *Methanonatronarchaeia*. In addition, the (reverse) methanogenesis marker gene *mcrA* has been repeatedly found outside *Euryarchaeota* in diverse environments, expanding the methanogenic metabolic potential in other archaeal phyla (Evans et al., 2019). *Bathyarchaeota* are some of the most dominant archaeal phyla in anoxic marine sediments and they are speculated to be important in degradation of diverse organic compounds as well as carbon cycling, potentially via methane metabolism (Zhou et al., 2018).

The methanogenic groups found in sediments depend greatly on physiochemical conditions such as salinity, pH, temperature, and available substrates (Webster et al., 2015). A global meta-analysis on biogeographical distribution of *mcrA* sequences showed that estuaries harbor the highest diversity of methanogenic lineages (Wen et al., 2017), potentially due to availability of various substrates of terrestrial and marine origin as well as a salinity gradient, which could create micro niches with ideal conditions for several lineages. Conventionally, methanogens are thought to inhabit and produce methane only in the deep methanic zones below the SMTZ via acetoclastic and hydrogenotrophic methanogenesis (Reeburgh, 2007), and this view is often supported by porewater methane profiles. However, vertical depth profiles of 16S rRNA gene sequences and MAGs combined with gas flux measurements and activity assays have questioned this view. Especially in eutrophic coastal sediments, methanogens are not necessarily outcompeted by SRB and may produce methane even close to the sediment-water interface due to a high abundance of both competitive (i.e., acetate) and non-competitive (i.e., methanol) substrates (Maltby et al., 2016; Zhuang et al., 2016; Sela-Adler et al., 2017; Kallistova et al., 2020). Therefore, methylotrophic methanogenesis may be an important but previously overlooked methane source in coastal shallow sediments (Conrad, 2020), and trimethylamine, dimethylsulfide, and dimethylsulfoniopropionate may be more common methanogenic substrates in marine sediments than currently thought (Zhuang et al., 2017), especially under higher salinity. Methylotrophic methanogens can co-exist with acetogens, SRB and ANME in OMZ sediments (Bhattacharya et al., 2020) and deep sea methane seeps (Li et al., 2020; Xu et al., 2020), and methanogenesis in the sulfate-reducing zone has been identified in coastal sediments (Maltby et al., 2016). However, whether these microorganisms compete for the same substrates or exist in separate niches remains unclear.

### Aerobic Methanotrophs

Aerobic MOB are widespread and active in marine ecosystems, with lineages that appear to thrive exclusively in marine environments, such as *Methylomicrobium japanense*-like organisms, and lineages deep sea-1 to -5 (Lüke and Frenzel, 2011). MOB have been detected in deep methane seepage sediments (Case et al., 2017), shallow hydrothermal systems (Hirayama et al., 2014), and in marine animals as symbionts (Petersen and Dubilier, 2009). MOB communities seem to differ between high methane seepage sediments and the overlying water column, the latter potentially hosting novel, poorly understood MOB lineages (Tavormina et al., 2008). In coastal areas of the Baltic Sea, aerobic methane oxidation rates in the water column achieve a maximum of 11.6 nmol L^–1^ d^–1^ over summer, when oxygen concentrations are lowest, highlighting that methanotrophs adapted to hypoxia may contribute significantly to mitigating methane emissions in such ecosystems (Steinle et al., 2017).

However, in coastal sediments, MOB seem to be present at low abundances. For instance, while in coastal sites of the Bothnian Sea only few Type I methanotrophic *Methylococcaceae* sequences were detected, the relative abundance of this group reached 6% of total bacterial reads at an offshore site (Rasigraf et al., 2020). Similarly, in coastal sediments of the Baltic Sea, the relative abundance and RNA transcripts attributed to the aerobic methanotrophy marker protein pMMO of the dominant methanotroph *Methylococcales* were significantly higher in deeper coastal offshore areas compared to adjacent shallow zones, which had higher surface water methane concentrations (Broman et al., 2020). The authors hypothesized that, in coastal sites, ebullition prevented the access of MOB to methane, limiting their growth, and that light might inhibit methanotrophic activity. Nonetheless, experimental oxygenation of Baltic Sea sediments resulted in increased MOB activity, as suggested by *pmoAB* RNA transcripts and 16S rRNA genes matching *Methylococcales* (Broman et al., 2017), indicating that MOB in coastal sediments may become active under favorable conditions. Similarly, MOB were found at maximum relative abundances of 0.012% in top sediments of the East China Sea coast, but were enriched after incubation with methane (He et al., 2019). Given that the most abundant MOB affiliated to the Type II methanotroph *Methylosinus*, which have a high affinity for methane, the authors hypothesized that these MOB could be surviving on “methane leftovers” after the activity of ANME and NC10 microorganisms. Moreover, MOB, at low abundances, may be found together with ANME in coastal sediments, suggesting potential effects of bioturbation (Myllykangas et al., 2020b).

Marine MOB may utilize alternative terminal electron acceptors. *Methylococcales* were identified as partially denitrifying methanotrophs in the OMZ of Gulfo Dulce via 16S rRNA gene analyses, metagenomics and metatranscriptomics (Padilla et al., 2017). It has been experimentally shown that aerobic MOB can couple methane oxidation to nitrate reduction under oxygen limitation (Kits et al., 2015). More recently, pure cultures of *Methylomonas* and *Methylosinus* were shown to couple methane oxidation to ferrihydrite reduction under oxygen limitation (Zheng et al., 2020). Such metabolisms are poorly investigated in marine ecosystems. Moreover, MOB in coastal sediments may have distinct biochemistry and be phylogenetically diverse. Some novel MOB isolated from North Sea sediments, including the first cultivated marine Alphaproteobacterial methanotroph, rely solely on a soluble methane monooxygenase for methane oxidation (Vekeman et al., 2016a), while others utilize a lanthanide-dependent XoxF5-type methanol dehydrogenase for methanol oxidation (Vekeman et al., 2016b). MOB affiliated to the phylum Verrucomicrobia (*Ca.* Methylacidiphilum) have been identified at low abundances in the coastal sediments of the Baltic Sea (Myllykangas et al., 2020b). Additionally, poorly classified Verrucomicrobia sequences have been identified in coastal sediments of the Bothnian Sea (Rasigraf et al., 2020), which were used for inoculation of a methane and iron-fed bioreactor that enriched Verrucomicrobia under oxygen limitation (Dalcin Martins et al., 2020). Further studies are needed to elucidate the roles as well as metabolic and phylogenetic diversity of MOB in coastal sediments.

### Identifying and Understanding Responses of Methane-Cycling Microbial Communities to Anthropogenic Disturbances

As the metabolic potential of most microorganisms is still not known, it is difficult to understand how complex biogeochemical cycles and microorganisms of coastal sediments are linked. Studies using 16S rRNA genes for phylogenetic analysis have shown a quite similar vertical diversity of bacterial and archaeal sequences both in freshwater (Borrel et al., 2012; Rissanen et al., 2019) and marine sediments (Petro et al., 2017; Rasigraf et al., 2020). The microbial diversity is highest in surface sediments, while it decreases in deeper layers, with only a few key taxa abundant in all layers. Methanogenic Euryarchaeota often dominate the deepest sediments, which harbor a large fraction of unclassified or less known taxa, such as Bathyarchaeota (Evans et al., 2015; Rissanen et al., 2019). The physiology of these recently identified archaea is poorly understood, and without cultivated representatives, phylogenetic studies do not tell us about the active metabolic pathways in the deep methanogenic zones.

Sediment redox zones can change rapidly upon variations in bottom water salinity, temperature, oxygen and nutrient concentrations and/or the input and composition of organic matter. While microbial activity in sediments is sensitive to these changes, the response could be delayed because of a lack of sufficient biomass for a given reaction (Dale et al., 2008a). In addition, bacterial and archaeal species may react to changes differently, e.g., archaea may be less sensitive to salinity changes but may respond to increases in organic carbon (Swan et al., 2010). Therefore, to estimate the effect of anthropogenic eutrophication and climate change on coastal methane emissions, the factors driving changes in the microbial community need to be understood. Increased hypoxia in eutrophic areas may have long-term effects on the microbiome, promoting methane emissions (Egger et al., 2016b). However, Broman et al. (2017) showed that restoration efforts for re-oxygenation may reverse increased methane fluxes. Metatranscriptomic analysis found elevated levels of transcripts for methanotrophic genes already one month after the return of oxic conditions in Baltic Sea sediment incubations.

Natural coastal areas are also affected by human-induced modifications, and, in recent years, many areas such as salt marshes and wetlands are being restored to their previous state. A microbiome investigation of salt marshes, habitats rich in organic matter and potential greenhouse gas sources, saw vast differences between the sediment microbiomes in nearby locations before and after restoration (Morris et al., 2019). In Jamaica Bay (United States), native salt marshes harbored higher taxonomic diversity than restored marshes. The younger the restored marsh was, the more distinct the bacterial communities were when compared to the microbiome of the natural, degraded marsh. In addition to loss of diversity in restored coastal sediments, Thomas et al. (2019) observed a loss of the SRB community in newly amended marshes in the US East coast. As sulfate reduction is the main pathway for organic matter degradation in salt marshes and an inhibitor/competitor of methanogenesis (Giani et al., 1996), the change in the microbiome might alter the metabolic pathways possibly resulting in increased methanogenesis in anoxic sediments below the oxic sediment layer. As the microbial community is very sensitive to changes in sediment redox zones, oxygenation, and organic matter composition, it is important to understand the consequences of restoration effects of different coastal areas. Even though reoxygenation may mitigate methane emissions via aerobic methane oxidation, restoring other areas to their natural state may disturb a stable microbial community and the functioning metabolic pathways, which may have unforeseen effects on the biogeochemical cycling and greenhouse gas emissions.

## Estimating Methane Emissions From Coastal Sediments

Eutrophication and climate change resulting from anthropogenic activity are strongly affecting coastal ecosystems, their biogeochemical functioning and microbiome. High rates of sedimentation combined with a high organic matter input, bottom water oxygen depletion and rising temperatures alter rates of organic matter burial and the electron acceptors available for its mineralization in coastal sediments. All of these processes affect methane production and oxidation. In order to mitigate emissions from coastal sediments, it is important to have a quantitative understanding of how methane-cycling processes respond to environmental change.

Whereas in offshore marine sediments methane emissions are regulated by a stable SMTZ (Weber et al., 2019), estimating methane sinks and sources across more complex coastal systems is challenging. Frequently, fluctuating hydrological conditions and seasonality affect sulfate concentrations, salinity, nutrient input and the type and abundance of organic matter, which all have an influence on methanogenic and –trophic processes.

Rapid sediment accumulation can be a cause for both increased methanogenesis and decreased AOM, as more labile organic carbon substances are buried below a shallow SMTZ with poorly constructed ANME consortia (Egger et al., 2016b). For instance, S-AOM can be replaced by Fe-AOM and/or N-AOM, which often occur in coastal sediments with low sulfate concentration (Egger et al., 2015b; Shen et al., 2016). ANME-2 archaea and NC10 bacteria responsible for N-AOM and Fe-AOM can also exist in the methanogenic zone and outcompete methanogens for their substrates, thereby mitigating the effects of increased methanogenesis. However, these methane oxidation pathways are regulated by the abundance of sulfate, methane, iron oxides and salinity, among many factors, and therefore the local conditions will determine their effectiveness.

Methanogens can use various substrates for methane production, thus the rate of methanogenesis can be difficult to estimate without knowing the sediment composition in the methanogenic layers (Reeburgh, 2007). Furthermore, in coastal sediments, methanogenic archaea and anaerobic methanotrophs can co-exist across the sediment profile. This leads to an overlap in redox zones, shoaling of the SMTZ and burial of more labile organic matter in the methanic zone (Rooze et al., 2016). In addition, the microbial community composition can play an important role, as ANME-1 may be capable of both methanogenesis and AOM, depending on the amount of hydrogen/methane being available (Kevorkian et al., 2020). All these interactions contribute to the methane cycle in sediments and make the potential processes difficult to predict.

Reactive transport models are useful in integrating geochemical data from field studies to quantify methane dynamics in sediments (e.g., Dale et al., 2008b; Regnier et al., 2011; Mogollón et al., 2012; Rooze et al., 2016). Reactive transport models can be used to reconstruct changes in sediment biogeochemistry and methane dynamics on time scales of decades (Rooze et al., 2016) to millennia (Mogollón et al., 2012; Egger et al., 2017). This can help to estimate how the effects of eutrophication will be propagated. Additionally, eutrophication can cause many local changes depending on hydrological conditions, oxygen concentration, water level and for example the location of sewage treatment plants close by Thang et al. (2013). These local differences can make the model predictions less reliable. Another factor making the model outcome less precise is seasonality. As shown in the Gulf of Mexico (Gelesh et al., 2016), methane emissions are affected by seasonal hypoxia, but many other factors such as sudden weather changes also affect the amount of methane released. Therefore, both spatial and temporal trends of methane emissions are difficult to capture by upscaling local data with reactive transport models. To allow the development of larger scale models, better recording of sediment profiles together with better understanding on the factors regulating the microbial methane production and oxidation in eutrophic conditions are needed.

Microorganisms play a critical role in controlling biogeochemical processes in sediments. At present, we cannot predict how these processes will respond to environmental change, because this may depend on the composition of the active microbial community. Gene-centric models, which are biogeochemical models that include an explicit representation of microbial processes, have the potential to greatly enhance our insights in coupled elemental cycling in sediments (Reed et al., 2014; Louca et al., 2016; Kreft et al., 2017). As the functional genes involved in methane production and oxidation are well known, meta-omics could be applied to target key genes and active metabolic processes. Combined with 16S rRNA gene analyses and geochemical profiles, these improved models could make better predictions of methane emissions from coastal sediments. To achieve this, a major focus of future research should be on understanding of the complex network of the microbial methane cycle and identifying the responsible microorganisms.

## Conclusion

Methane cycling in coastal environments is a complex process with many factors regulating both methane production and oxidation. Anthropogenic eutrophication and climate change are greatly impacting coastal ecosystems and are likely enhancing methane emissions from these environments in the future. Rapid sedimentation rates, bottom water oxygen depletion and rising temperatures can all contribute to increased methane production in coastal sediments. Further increased organic matter inputs and lower salinity reduce the rates of S-AOM which decreases the methane-filtering potential of coastal sediments. Influx of nutrients, especially nitrate and metal oxides on the other hand can serve as electron acceptors for alternative AOM pathways Taken together the consequences of eutrophication and climate change most likely will increase overall methane emissions. Establishing better gene-centric models including the activity of the responsible microorganisms will be an important study area to better understand and predict the microbial methane cycle in coastal systems.

## Author Contributions

AW collected the data and literature, wrote the draft version, and composed the tables and figures. PDM, CS, and MJ critically reviewed and discussed the subsequent versions, and rewrote several sections. All authors contributed to the article and approved the submitted version.

## Conflict of Interest

The authors declare that the research was conducted in the absence of any commercial or financial relationships that could be construed as a potential conflict of interest.
